# Cross-reactive MHC class I T cell epitopes may dictate heterologous immune responses between respiratory viruses and food allergens

**DOI:** 10.1038/s41598-023-41187-1

**Published:** 2023-09-08

**Authors:** Kathrin Balz, Abhinav Kaushik, Franz Cemic, Vanitha Sampath, Vanessa Heger, Harald Renz, Kari Nadeau, Chrysanthi Skevaki

**Affiliations:** 1 Institute of Laboratory Medicine, Universities of Giessen and Marburg Lung Center (UGMLC), Philipps University Marburg, German Center for Lung Research (DZL), 35043 Marburg, Germany; 2 Division of Pulmonary, Allergy and Critical Care Medicine, Sean N. Parker Center for Allergy and Asthma Research at Stanford University, Stanford, CA 94040 USA; 3 Departmental of Environmental Health, Harvard T. H. Chan School of Public Health, Boston, MA 02115 USA; 4 Department of Computer Science, TH Mittelhessen, University of Applied Sciences Gießen, 35390 Giessen, Germany

**Keywords:** MHC class I, T cells, Allergy, Asthma

## Abstract

Respiratory virus infections play a major role in asthma, while there is a close correlation between asthma and food allergy. We hypothesized that T cell-mediated heterologous immunity may induce asthma symptoms among sensitized individuals and used two independent in silico pipelines for the identification of cross-reactive virus- and food allergen- derived T cell epitopes, considering individual peptide sequence similarity, MHC binding affinity and immunogenicity. We assessed the proteomes of human rhinovirus (RV1b), respiratory syncytial virus (RSVA2) and influenza-strains contained in the seasonal quadrivalent influenza vaccine 2019/2020 (QIV 2019/2020), as well as SARS-CoV-2 for human HLA alleles, in addition to more than 200 most common food allergen protein sequences. All resulting allergen-derived peptide candidates were subjected to an elaborate scoring system considering multiple criteria, including clinical relevance. In both bioinformatics approaches, we found that shortlisted peptide pairs that are potentially binding to MHC class II molecules scored up to 10 × lower compared to MHC class I candidate epitopes. For MHC class I food allergen epitopes, several potentially cross-reactive peptides from shrimp, kiwi, apple, soybean and chicken were identified. The shortlisted set of peptide pairs may be implicated in heterologous immune responses and translated to peptide immunization strategies with immunomodulatory properties.

## Introduction

During the last few decades, the prevalence of allergic diseases has dramatically increased in developed countries. The incidence of asthma has increased fourfold since the 1950’s^1^ and food allergy prevalence among children has increased to 3.5–8%^2^. Food allergy is often classified into either IgE-mediated, non-IgE-mediated, or mixed IgE/non-IgE-mediated allergic disease. Non-IgE-mediated food allergy is thought to be initiated by T cells, although the pathophysiological mechanisms underlying these reactions are not yet fully understood^3, 4^. Studies have observed that food allergy is associated with subsequent increases in the development of allergic rhinitis and asthma^5^. Both consumption and inhalation of food allergens can cause allergic reactions in sensitized individuals. Inhalation of aerosolized wheat, lupin, and other food allergens are thought to stimulate mast cells in the lung and have been associated with respiratory symptoms, including wheeze^6–8^. As wheezing in childhood is mostly associated with viral infections, an indirect correlation between viral infections and food allergy has been suggested. Gastrointestinal viral infections are also more relevant in the context of food allergy^9^. Some mouse studies provide evidence for the subsequent development of food allergen specific IgE on exposure to food allergens after gastrointestinal infection with certain RNA viruses, including murine norovirus type 1 or reovirus^10, 11^.

We have previously shown an influenza virus-mediated protective effect over development of experimental asthma in models of ovalbumin and house dust mite-induced asthma. This effect was mediated by cross-reactive T effector memory cells^12^. We hypothesized that cross-reactive T cell epitopes present in respiratory viruses and food allergens may provide the missing link between allergy and wheeze.

Therefore, our aim was to identify potentially cross-reactive T cell epitope pairs among food allergens and clinically relevant respiratory viruses. Such data can serve as the basis for investigating heterologous immune responses among patients with a specific food allergy and a viral infection or even for following outcomes (beneficial or harmful) of antiviral vaccination.

Recently, computational advances have significantly improved our understanding of cross-reactive sites across different allergens. These methods screen for antigenic peptides with similar epitope profile to bind a particular T cell receptor (TCR)^13^. However, most of these methods have their input dataset requirements (e.g., TCRseq or expression data or 3D structure of proteins) and with limited or no options to customize the source of training dataset (e.g., training with experimentally validated T-cell epitopes only) to achieve case-specific objectives^14–16^. Therefore, in this study, we applied two independent approaches developed in-house to predict MHC binding cross-reactive peptide sequences across viral and food allergen sequences that can potentially cause similar T-cell response. These tools also include prediction of MHC binding affinity, which is highly dependent on the so called “anchor residues” of the peptides. Such residues represent at least two amino acids, which are exactly fitting into the groove of the MHC molecule. Thus, anchor residues play an important role in the definition of antigenic peptides^17^.

## Results

We used partially modified versions of our previously published in-silico pipelines for prediction of potentially cross-reactive T cell epitope pairs between allergens and viruses, focusing on food allergens and clinically relevant respiratory viruses. The pipeline-1 uses an agnostic approach to predict the antigenic peptides by means of T cell epitope prediction for both viruses and allergens as well as for the sequence homology of the predicted epitopes, whereas pipeline-2 uses a supervised approach, wherein IEDB T-cell epitope repertoire was used to predict the cross-reactive antigenic regions across viruses and allergens.

**Figure 1:**
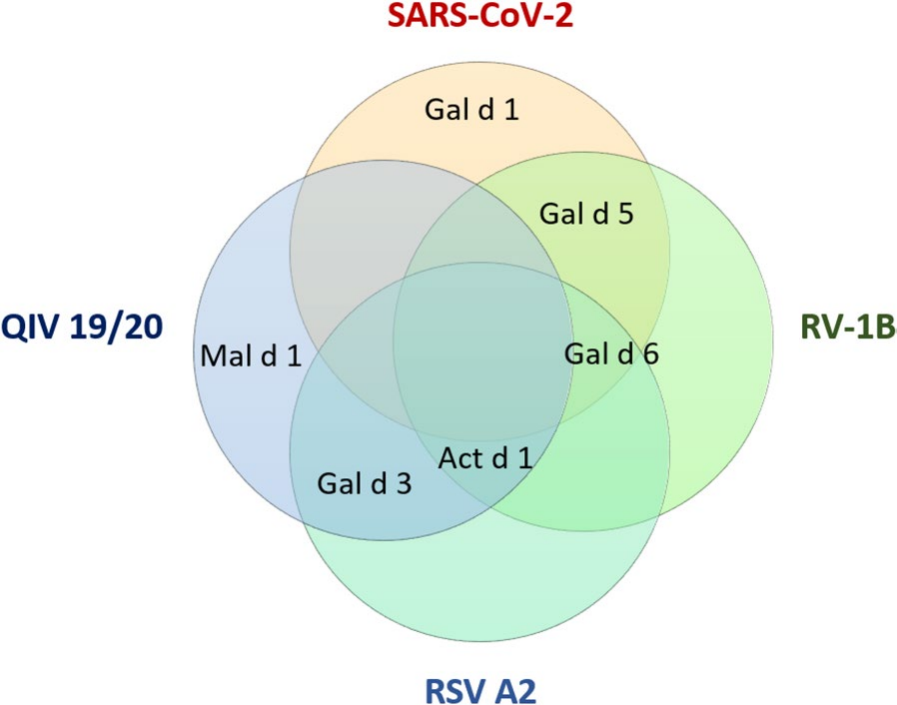
Venn diagram depicting the molecular allergen components containing the allergen counterpart of the top 5 predicted epitope pairs for HLA class I prediction with pipeline-1. The top 5 candidates were predicted with pipeline-1 and ranked based on the described scoring system. Molecular allergen components contained in the predicted top 5 allergen epitopes for each virus are depicted in the Venn diagram.

Pipeline-1 predicted multiple potentially-cross-reactive T cell epitope pairs for each virus, which were further ranked based on the calculated pair combined score and subsequently scored for the top 5 candidate pairs based on additional criteria. Among the top 5 results for all viruses, we identified T cell epitope pairs with the allergenic epitopes deriving from kiwi (*Actinidia* *deliciosa,* *Act* *d*), chicken (*gallus* *gallus,* *Gal* *d*) and apple (*malus* *domestica,* *Mal* *d*) (Fig. 1). The top 5 candidates for RSVA2, RV1b and influenza strains of the seasonal quadrivalent influenza vaccine 2019/2020 (QIV 19/20) on the background of the most frequent human HLA class I alleles are depicted in Table 1. The corresponding top 5 candidate pairs for SARS-CoV-2 for pipeline-1 were published previously^18^.

**Table 1:** Top 5 candidate epitope pairs for RV1b, RSVA2 and Influenza vaccine 2019/2020 on the background of the most frequent human HLA alleles (Supplementary Table 3), based on pipeline-1 and calculation of the final score as described (see “Materials and methods”).

	Allergen epitope	Allergen	Allergen protein family	Viral epitope	Viral protein	MHC allele	Final score	
A: RV1b								
Nr. 1	ASDVICQEY	Gal d 5	Serum albumin	MHDSILVSY	P2-A	HLA-A*01:01	1258	
Nr. 2	VSDDGLNIY	Gal d 6	Lipoprotein	LSCKFLPLY	P2-C	HLA-A*01:01	1032	
Nr. 3	FADLTNEEY	Act d 1	Papain-like cysteine protease	YIPETEDDY	Protease	HLA-A*01:01	610	
Nr. 4	KVFRFSMFK	Gal d 6	Lipoprotein	GIFGENMYY	VP2	HLA-A*11:01	508	
Nr. 5	FLGDVIPPGI	Gal d 6	Lipoprotein	LLLAYTPPGI	VP3	HLA-A*02:01	484	
B: RSVA2								
Nr. 1	FLGDKFYTV	Gal d 3	Transferrin	FLPDKISLT	RNA-directed RNA polymerase L	HLA-A*02:01	1166	
Nr. 2	VYMDLPHGI	Mal d	Unknown	LYMNLPMLF	RNA-directed RNA polymerase L	HLA-A*24:02	1148	
Nr. 3	TTYKEFLGDK	Gal d 3	Transferrin	TTYNQFLTWK	RNA-directed RNA polymerase L	HLA-A*11:01	642	
Nr. 4	FADLTNEEY	Act d 1	Papain-like cysteine protease	KSNRYNDNY	RNA-directed RNA polymerase L	HLA-A*01:01	586	
Nr. 5	YLLDLLPAA	Gal d 6	Lipoprotein	YLSELLNSL	RNA-directed RNA polymerase L	HLA-A*02:01	516	

	Allergen epitope	Allergen	Allergen protein family	Viral epitope	Virus protein	Virus strain	MHC allele	Final score
C: Influenza vaccine 2019/2020								
Nr. 1	SPARLYNAL	Mal d 1	Bet v 1 family	NPAMLYNKM	PB2	A/Kansas/2017	HLA-B*07:02	1784
Nr. 2	YLLQNPEAYV	Mal d 1	Bet v 1 family	SLYQNADAYV	HA	A/Brisbane/2018	HLA-A*02:01	636
Nr. 3	FLGDKFYTV	Gal d 3	Transferrin	FMYSDFHFI	PA	A/Brisbane/2018, A/Kansas/2017	HLA-A*02:01	604
Nr. 4	KPTSKRMAI	Act d 1	Papain-like cysteine protease	EPESKRMSL	NS1	B/Colorado/2017, B/Phuket/2013	HLA-B*07:02	556
Nr. 5	YTQTYGVDY	Mal d	Unknown	YTDTYHSYA	NA	B/Colorado/2017, B/Phuket/2013	HLA-A*01:01	432

Interestingly, sequences from apple only appeared in the QIV/19/20 top 5 candidate epitope sequences. Sequences from Gal d 6 and Act d 1 were predicted in 3 out of the 4 virus analyses, suggesting an important role in cross-reactivity between respiratory viruses and food allergens. With regards to the viral proteins, the predicted top 5 sequences for RSVA2 were derived exclusively from the RNA-directed polymerase, whereas the viral protein source for the other viruses were more diverse. Of note, when applying the pipeline for human HLA class II prediction, no candidate epitope pairs could be identified for RV1b and QIV19/20. Epitope pairs for RSVA2 achieved similar scores in both HLA class I and II analyses (Table 2). Importantly, such epitopes derived from a variety of viral proteins and different allergenic sources compared with candidates for HLA class I. Allergenic sources include buckwheat (*Fagopyrum* *esculentum,* *Fag* *e*), sesame (*Sesamum* *indicum,* *Ses* *i*), potato (*Solanum* *tuberosum*, *Sola* *t*), and hazelnut (*Corylus* *avellana,* *Cor* *a*), with almost all belonging to the protein family of cupins.

**Table 2:** Top 5 candidate pairs for RSVA2 on the background of the most frequent human HLA class II alleles (Supplementary Table 3) based on pipeline-1 and calculation of the final score as described (see “Materials and methods”).

	Allergen epitope	Allergen	Allergen protein family	Viral epitope	Viral protein	MHC allele	Final score
1	Fag e 1	LPILEFLQLSAQHVV	Cupin	KGAFKYIKPQSQFIV	Matrix protein	HLA-DRB1_01_01	1292
2	Ses i 3	VLFALLLASAVVASE	Cupin	MELLILKANAITTIL	Fusion glycoprotein F0	HLA-DRB1_01_01	666
3	Cor a 9	ARRLKYNRQETTLAR	Cupin	LRWLTYYKLNTYPSL	RNA-directed RNA polymerase L	HLA-DRB1_04_01	638
4	Sola t 1	FAKLLSDRKKLRANK	Patatin family	TELNSDDIKKLRDNE	Matrix M2-1	HLA-DRB1_03_01	350
5	Fag e 1	LPILEFIQLSAQHVV	Cupin	KGAFKYIKPQSQFIV	Matrix protein	HLA-DRB1_01_01	168

In order to validate our in-silico results we applied an independent pipeline that uses known T cell epitope features as they appear on IEDB tools. The pipeline is an extension of a computational framework we published previously^18^, wherein similarity between identical *k*-mers with experimentally validated T cell epitopes was used to predict cross-reactive peptides. In this analysis, we observed a limited number of cross-reactivity sites between food and viral antigens, wherein, the largest number of cross-reactive peptides were predicted between *Glycine* *max* (soybean) and SARS-CoV-2 replicase polyprotein. Besides the polyprotein, SARS-CoV-2 spike glycoprotein also shares potential cross-reactive amino acids with the sarcoplasmic ca-binding proteins of *Crangon* *crangon* (shrimp)*.* In addition to SARS-CoV-2, we also observed that human respiratory syncytial virus A proteins also share potential cross-reactive sites with food allergen sequences from *Crangon* *crangon* and *Malus* *domestica* (apple). Overall, six different protein sequences from two viral species were found to share cross-reactive amino acids with three food allergens. Wherein, we predicted 44 redundant (16 non-redundant) cross-reactive peptide pairs between those six viral sequences with 16 different food allergen sequences (Table 3; Supplementary Table 3). A total of 22 unique peptides from food and viral allergens were finally shortlisted that were predicted to bind 27 unique class-I HLA alleles, that includes both HLA-A and HLA-B genes.

**Table 3:** Cross-reactive peptide peptides predicted between viral and allergen sequences using pipeline-2.

Virus name	Protein	Viral sequence	Allergen	Protein	Allergen sequence
SARS-CoV2	Replicase_polyprotein	RLQAGNATEVPANST	*Glycine* *max*	Beta-conglycinin protein	LQSGDALRVPAGTTF
SARS-CoV2	Spike_glycoprotein	GINITRFQTLLALHR	*Glycine* *max*	Beta-conglycinin protein	LQSGDALRVPAGTTY
SARS-CoV2	Spike_glycoprotein	LPIGINITRFQTLLA	*Glycine* *max*	Beta-conglycinin protein	LQSGDALRVPAGTTY
SARS-CoV2	Replicase_polyprotein	DEGNCDTLKEILVTY	*Crangon* *crangon*	Iosephosphate isomerase	PCIGEKLDERESNRT
SARS-CoV2	Replicase_polyprotein	LQAGNATEVPANSTV	*Crangon* *crangon*	Iosephosphate isomerase	PCIGEKLDERESNRT
HRSV A (strain A2)	RNA-directed RNA polymerase L	RLMEGQTHAQADYLL	*Malus* *domestica*	Predicted protein (E4Z8N9)	RLFARTRQVESLTAE
SARS-CoV2	Replicase_polyprotein	RLQAGNATEVPANST	*Malus* *domestica*	Predicted protein (E4Z8N9)	RLFARTRQVESLTAE
SARS-CoV2	Replicase_polyprotein	RLQAGNATEVPANST	*Malus* *domestica*	Putative COBL7 (COBRA-LIKE 7)	RLFARTRQVESLAAE
HRSV A (strain A2)	Non-structural protein 2	CIVRKLDERQATFTF	*Crangon* *crangon*	Sarcoplasmic calcium-binding protein	AGGINIARYQELYAQ
SARS-CoV2	Replicase_polyprotein	DEGNCDTLKEILVTY	*Crangon* *crangon*	Sarcoplasmic calcium-binding protein	GINIARYQELYAQFI
HRSV A (strain A2)	Non-structural protein 2	CIVRKLDERQATFTF	*Crangon* *crangon*	Sarcoplasmic calcium-binding protein	KAGGINIARYQELYA
SARS-CoV2	Membrane protein	RLFARTRSMWSFNPE	*Crangon* *crangon*	Sarcoplasmic calcium-binding protein	AGGINIARYQELYAQ
SARS-CoV2	Spike_glycoprotein	PIGINITRFQTLLAL	*Crangon* *crangon*	Sarcoplasmic calcium-binding protein	GGINIARYQELYAQF
SARS-CoV2	Membrane protein	RLFARTRSMWSFNPE	*Crangon* *crangon*	Sarcoplasmic calcium-binding protein	GINIARYQELYAQFI
SARS-CoV2	Replicase_polyprotein	DEGNCDTLKEILVTY	*Gallus* *gallus*	Serum albumin	DHGEADFLKSILIRY
SARS-CoV2	Spike_glycoprotein	GINITRFQTLLALHR	*Gallus* *gallus*	Serum albumin	DHGEADFLKSILIRY
SARS-CoV2	Spike_glycoprotein	GINITRFQTLLALHR	*Malus* *domestica*	Two-component response regulator	MKGVTHGACDYLIKP

Comparing the T cell epitope pairs predicted with both pipelines, no exact sequences were predicted commonly, due to the differences in peptide length and algorithms used by the developed pipelines. However, predicted allergenic sequences from *malus* *domestica* and *gallus* *gallus* were identified with both pipelines and sequences from *gallus* *gallus* were even predicted to potentially cross-react with viral epitopes from SARS-CoV-2 by both approaches.

## Discussion

We identified several potentially cross-reactive T cell epitope pairs between food allergens and epidemiologically relevant respiratory viruses, using two independent in-silico pipelines. To our knowledge, this is the first study investigating heterologous immunity between T cell epitopes among several clinically relevant RNA viruses and food allergens. Our study revealed allergenic sequences from *malus* *domestica* to be important for cross-reactivity. Further, we found that the epitope sequences from SARS-CoV-2 and RSVA2 were those predicted to most likely cross-react with food allergens, as T cell epitope pairs were predicted by both pipelines. Finally, we observed more candidate epitope pairs for HLA class I compared to HLA class II.

In pipeline-1, we made use of several T cell epitope prediction tools followed by alignments based on sequence homology and amino acid properties. The latter is particularly important for detection of cross-reactivity, which is known to also exist between structurally unrelated antigens with little sequence homology^19^. In addition, it is known that the position of specific amino acids in their non-anchor region is critical for T cell epitope immunogenicity^20^. A limitation of pipeline-1 is the lack of such tools for prediction of immunogenicity, which is compensated by pipeline-2. Indeed, the latter utilized the IEDB immunogenicity prediction tool for this purpose^20^.

We applied pipeline-2 to identify only known T cell epitopes in combination with alignment against respiratory bacteria in order to avoid prediction contaminations. Hence, this restriction led to the lack of any identified epitope pairs for RV1b and influenza strains containing the seasonal quadrivalent influenza vaccine. The allergen sequences among the short-listed epitope pairs for SARS-CoV-2 and RSVA2 were derived from different allergen sources, including apple and chicken. Importantly, using pipeline-1, several candidate epitope pairs involving SARS-CoV-2 contained chicken allergens as published previously^18^.

In our study, we did not observe identical peptide sequence pairs, commonly predicted by both applied pipelines. The reason for non-overlapping sequence pairs is the use of independent sequence features by the applied pipelines such as associated immunogenicity and peptide lengths. Moreover, several predicted peptide pairs have not been previously reported and may represent novel sets of epitopes. Overall, these two independents algorithms predicted an exhaustive set of non-redundant epitope pairs that require further experimental investigation.

Of note, we also predicted epitope pairs for RSVA2 on the background of human HLA class II alleles. Interestingly, no candidate pairs could be identified using RV1b or influenza proteins. In addition, we were able to identify candidate pairs for SARS-CoV-2 but with a 10 × lower score compared to HLA class I-restricted epitopes^18^. However, no HLA class II candidates were predicted with pipeline-2.

Aerosolized food allergens may trigger asthma symptoms via inhalation and subsequent inflammatory response in the lung^7^. Such cases have been shown for fish allergens (33) and soy proteins^21^. Importantly, we predicted T cell epitope sequences from RSVA2 and SARS-CoV-2 which appeared to be cross-reactive with sequences from *Crangon* *crangon,* a shrimp, as well as sequences from RSVA2 which showed cross-reactivity with sequences from soybean.

Our study provides *in-silico* data, which support a yet unexplored pathogenic mechanism for the connection between food allergy and virus-associated asthma. T cell-mediated heterologous immune responses are an important determinant for the final outcome of an infection or allergen exposure and can lead to protective responses or immunopathology, also depending on the balance between antigen load and efficiency of effector T cells. Immunomodulatory effects can thus be elicited by cross-reactive antigens, which may lead to an expansion of T memory cells and in turn to a modified T cell memory pool, changes in patterns of immunodominance and an altered hierarchy of T cell responses^22^. The final outcome depends not only on private specificities of TCR repertoires but also on the host’s HLA background.

Future studies using PBMCs from individuals with relevant food allergies may be the first step to validate cross-reactivity involving the predicted T cell epitope pairs. In order to address the diversity of prevalent HLA alleles in distinct ethnic populations, ideally experiments should involve pools of most frequently identified potentially cross-reactive peptides as well as large groups of individuals with distinct HLA haplotypes. Results of such studies may advise peptide immunization strategies for a favorable outcome in the context of allergy and infection.

## Materials and methods

We used our previously published in-silico analysis for prediction of potentially cross-reactive T cell epitope pairs between viral and allergenic proteins, applying two independent pipelines. We downloaded all available food allergens from Allergen Online (10.09.2017)^23–25^, as well as protein sequences from the most clinically relevant respiratory viruses SARS-CoV-2, RSVA2, RV1b and influenza strains of the seasonal quadrivalent influenza vaccine 2019/2020 (QIV19/20) (Uniprot and GISAID). Protein sequences are provided in Supplementary Tables 1 and 2.

Pipeline-1 was performed as described previously^18^, using highly prevalent human HLA alleles for Caucasian populations (Supplementary Table 3). Briefly, viral T cell epitopes were predicted using smm^26^, ann^27^ and consensus^28^ algorithm tools for MHCI (IC50 threshhold < = 5000 nm), and netMHCII^29^ for MHCII. Allergenic proteins were aligned against predicted viral epitopes (NCBI protein blast platform)^30^ and subsequently used for T cell epitope prediction by netMHC^31^ and netMHCpan^32^ for MHCI, and netMHCII and netMHCIIpan^33^ for MHC class II prediction. Viral and allergen epitopes predicted by all methods were pairwise aligned with a Biopython module pairwise 2^34^. A final pair combined score was calculated, taking the binding affinity of predicted viral and allergen epitopes to MHC molecules into consideration, as well as the score from the pairwise alignment and cross-entropy ($Pair \; \mathrm{ Combined \; Score}=\frac{1}{binding \; affinity \left(nM\right) \left(Virus\right)}\times score \; PwA\times \frac{1}{binding \; affinity \left(nM\right) (Allergen)}$). Allergenic epitopes of the top 30 candidate pairs further underwent a comprehensive scoring system taking clinical relevance and conservation criteria into consideration. Epitope pairs were finally ranked for the top 5 candidates for each virus based on the final score. A schematic overview of the pipeline is depicted in Supplementary Fig. 1A. The scoring system is shown in Supplementary Fig. 2.

In addition to the above pipeline-1, we also performed an analysis using a modified version of the independent pipeline we published previously (Supplementary Fig. 1B). We used IEDB database, which hosts known epitope peptide sequences, to predict peptides that are known to bind MHC molecules. To identify cross-reactive antigenic peptides between the given food allergenic protein sequences and viral proteomes, we split each of a given protein sequence into a set of sequential *k-mers* or peptides (length = 15). To avoid contaminations in the downstream analysis, we filtered out the sequential *k-mers* that mapped with bacterial protein sequences, using blastp (e-value < 1 and identity > 70% and coverage > 70%). Here, eight bacterial species (*Moraxella* *catarrhalis,* *Chlamydophila* *pneumoniae,* *Mycoplasma* *pneumoniae,* *Coxiella* *burnetii,* *Streptococcus* *pneumoniae,* *Haemophilus* *influenzae,* *Streptococcus* *pyogenes* and *Legionella* *pneumophila*) were selected that are known to cause common respiratory infections. Subsequently, using the IEDB immunogenicity prediction tool^20^, we identified from the remaining sequential *k*-mers those that can potentially form peptide MHC (pMHC) complexes. This tool uses the properties of amino acids within a given peptide to predict its potential to form pMHC complexes. The above steps filtered out a large proportion of sequential *k*-mers to retain only 134,171 high confidence peptide sequences. However, it is likely that some of the peptides may not have a strong MHC-I binding affinity despite the homology, and therefore may be less likely to be presented as antigens by HLA molecules. Therefore, using IEDB tools^35^, the homologous peptides were further evaluated for their binding affinity with human MHC-I molecules for a broad range of alleles (*n* = 54) (Supplementary Table 4a,b). The peptides with MHC binding affinity rank > 50 (arbitrarily selected threshold to retain at most only 50% of the total number of sequences) were further selected for homology analysis. Wherein, using the blastp (e-value < 1 and identity > 50% and coverage > 70%), we identified food allergen- and virus- peptides that share homologous sequences (Supplementary Table 4c) and which were considered as potentially cross-reactive T cell epitopes. All utilized algorithms/prediction tools in this study were accessed online rather from a local server.

We evaluated the statistical significance of predicted peptides (from method-2) by comparing the cross-reactive peptides with 1000 randomly generated sequences using BLASTp program. The random peptide sequences were generated using RSAT webserver (http://rsat.sb-roscoff.fr/random-seq_form.cgi). We measured the statistical significance, by calculating the p-value, as the number of times each of the predicted cross-reactive peptide matches with randomly generated sequences, using following equation.

$$p=n/1000.$$

Here, *n* = number of times a cross-reactive peptide shares sequence identity > 30% with random peptide sequences and e-value < 1.0 with the set of 1000 random peptide sequences. P-value < 0.05 was considered as statistically significant, to support alternative hypothesis that cross-reactive peptide sequence is significantly different from random sequences. Here, the low sequence identity threshold was used to filter out any cross-reactive peptide that matches with randomly generated peptide sequences.

### Supplementary Information

Supplementary Information 1.

Supplementary Information 2.

Supplementary Information 3.

Supplementary Information 4.

Supplementary Information 5.

## Data Availability

All data generated or analysed during this study are included in this published article and its supplementary information files.
