# Supplementary material for: Cross-reactive MHC class I T cell epitopes may dictate heterologous immune responses between respiratory viruses and food allergens
Source: Sci Rep. 2023 Sep 8;13:14874. doi: 10.1038/s41598-023-41187-1 (PMC10491592; doi:10.1038/s41598-023-41187-1)
Supplement: Supplementary file 1 — Supplementary Information 1. [file 41598_2023_41187_MOESM1_ESM.pdf]

Supplementary information

Homologies between respiratory viruses and food allergens may direct T cell-mediated heterologous immune responses

Kathrin Balz[1]*, MSc, Abhinav Kaushik[2]*, PhD, Franz Cemic[3], PhD, Vanitha Sampath[2], Vanessa Heger[3], BSc, Harald Renz[1], MD, Kari Nadeau[2], MD, PhD, Chrysanthi Skevaki[1], MD

[1]Institute of Laboratory Medicine, Universities of Giessen and Marburg Lung Center (UGMLC), Philipps University Marburg, German Center for Lung Research (DZL), Marburg, Germany

[2]Sean N. Parker Center for Allergy and Asthma Research at Stanford University and Division of Pulmonary, Allergy & Critical Care Medicine, Stanford, CA, USA.

[3] TH Mittelhessen, Department of Computer Science, University of Applied Sciences Gießen,

*equal contribution

CORRESPONDING AUTHOR

PD Dr. Chrysanthi Skevaki

Institute of Laboratory Medicine and Pathobiochemistry

Molecular Diagnostics

Philipps University Marburg

Baldingerstr, 35043 Marburg

Tel: +49 6421 5863850

Fax: +49 6421 5865594

Chrysanthi.Skevaki@uk-gm.de

Supplementary Figures

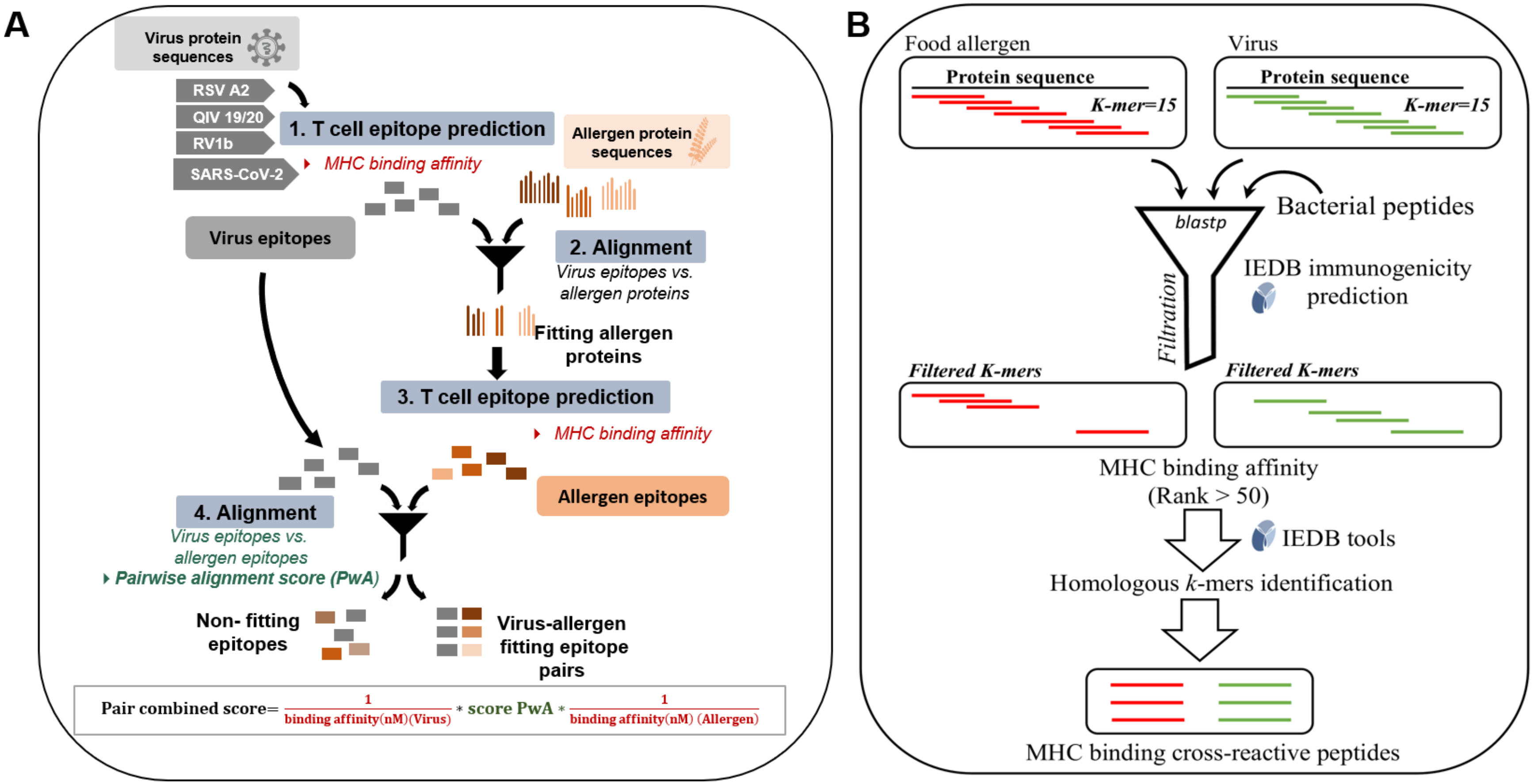

Suppl. Figure 1:
Schematic representation of the *in-silico* pipelines
Two independent in-silico pipelines were used for prediction of potentially cross-reactive T cell epitope pairs between food allergens and respiratory viruses. **A:** Pipeline-1 includes T cell epitope prediction for the virus and allergenic proteins which are used for pairwise alignment. A pairwise alignment score is calculated. **B**: Pipeline-2 uses kmers of virus and allergen protein sequences for alignment against bacterial peptides. Filtered kmers are finally aligned to identify MHC binding cross-reactive peptides. (adapted from Balz K. et al., SciRep, 2021)

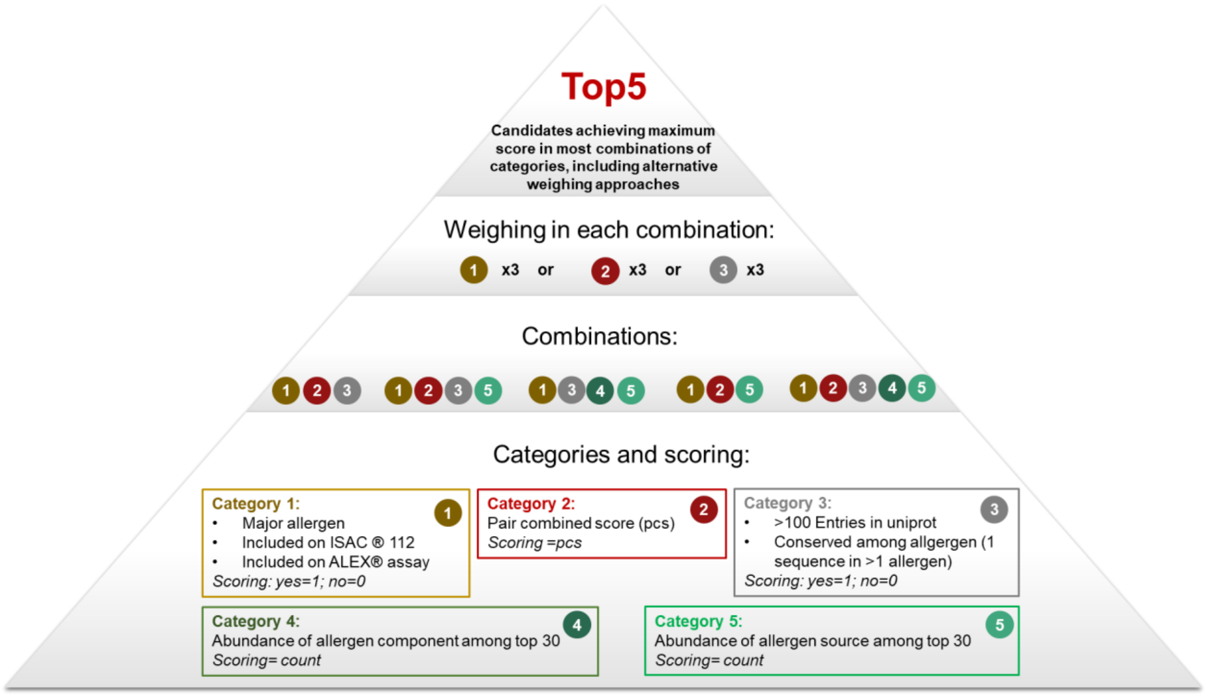

Suppl. Figure 2. Additional scoring system for further analysis of the top 30 potentially cross-reactive T cell epitope pairs of pipeline-1.

The top 30 candidate epitope pairs, as per pair combined score, were further assessed. A scoring system was developed and five categories of criteria were formed, summing up the scores of individual criteria in one group based on (1): whether they are included in the commercially available diagnostic assays ImmunoCAP ISAC®112 or ALEX® and evoked IgE production in >50 % of patients with associated clinical allergy as per WHO/IUIS Allergen Nomenclature Sub-committee (http://allergen.org/index.php) (2): pair combined score; defined in 4 ranges, with the help of percentiles: ≤ 10 % = 0; [20-50 %] = 1; [50-90 %] = 2; [90-100 %] (3): whether they achieve more than 100 entries when entering the epitope sequence in Uniprot and whether the specific epitope sequence is also contained in proteins of other allergen sources (4): abundance of the allergen component within the top 30 candidates (5): abundance of the allergen source within the top 30 candidates. As a next step, five different combinations of the aforementioned categories were defined and the cumulative score was calculated for each allergen epitope. Additionally, the cumulative score was calculated three more times, each time multiplying another category by a factor of 3 in order to critically compare alternative weighing of the associated criteria. The new top 5 candidate epitope pairs were subsequently ranked based on the frequency of achieving the maximum score in each of the separately weighed scoring approaches as described above.

(adapted from Balz K. et al., SciRep, 2021)

Supplementary tables

Supplementary Table 3: Human HLA alleles used for pipeline-1 (adapted from Balz K. et al., SciRep, 2021)

HLA Class I[1]	HLA II[2]
**HLA-A*01:01**	**DRB1*01:01**
**HLA-A*02:01**	**DRB1*03:01**
**HLA-A*11:01**	**DRB1*04:01**
**HLA-A*24:02**	
**HLA-B*07:02**	
**HLA-B*40:02**	

1=epitopes with a length of 9 or 10 amino acids

2=epitopes with a length of 15 amino acids
